# HDR-VDP-2, LPIPS, DISTS, and SSIM for Evaluating Computer-Generated Renderings: A Systematic Review of Use and Perceptual Validation

**DOI:** 10.3390/jimaging12100500

**Published:** 2026-10-10

**Authors:** Matija Grašić, Andrija Bernik, Vladimir Cviljušac

**Affiliations:** 1 University of Zagreb Faculty of Graphic Arts, Getaldićeva ulica 2, 10000 Zagreb, Croatia; mgrasic@unin.hr (M.G.); vladimir.cviljusac@grf.unizg.hr (V.C.); 2 Department of Multimedia, University North, 42000 Varaždin, Croatia

**Keywords:** computer-generated imagery, rendering, image quality assessment, perceptual metrics, photorealism, neural rendering, HDR-VDP-2, LPIPS, DISTS, SSIM

## Abstract

This systematic review examined the use and perceptual validation of four full-reference image quality metrics—HDR-VDP-2, LPIPS, DISTS, and SSIM—in computer-generated and rendered imagery. Among 283 included studies, SSIM was reported in 228 (80.6%) and LPIPS in 147 (51.9%), whereas HDR-VDP-2 and DISTS appeared in four each (1.4%). Only 23 studies (8.1%) included relevant human or subjective evaluation, and 13 (4.6%) directly tested metric–human agreement. Neural rendering, novel-view synthesis, and 3D reconstruction accounted for 175 studies (61.8%). Metric–human agreement varied with content, artifact type, alignment, viewing conditions, and perceptual task, while cue-specific evidence was uneven. The review therefore identifies a clear gap between metric adoption and perceptual validation. Metric selection should be matched to the rendering attribute, reference image, viewing conditions, and perceptual question.

## 1. Introduction

Computer-generated imagery (CGI) is increasingly evaluated not only by computational efficiency or physical correctness but also by the visual quality of the resulting image. This distinction is important when rendering algorithms approximate light transport, reduce sampling cost, reconstruct unseen viewpoints, or reduce computation at the cost of visually negligible differences. In such cases, an objective image quality metric is useful only if the measured difference is meaningfully related to what an observer can see or judge. Perceptual and visual-difference metrics therefore provide a practical way to compare rendered outputs, but their numerical values should not automatically be interpreted as measures of perceived quality or photorealism [1].

The four metrics considered in this review embody different assumptions about similarity. SSIM compares local luminance, contrast, and structural information and was proposed as an alternative to independent pixel-error measures [2]. HDR-VDP-2 explicitly models properties of the human visual system, including luminance-dependent contrast sensitivity and visual masking, to predict difference visibility and quality across luminance conditions [1]. LPIPS measures distances between deep feature representations and was developed from evidence that learned features can align more closely with human perceptual similarity than conventional pixel distances [3]. DISTS combines deep structural correlations with texture statistics and is designed to tolerate some texture resampling that may be numerically large but perceptually acceptable [4]. Their scores therefore represent different perceptual constructs rather than interchangeable estimates of realism.

The four metrics were selected as a deliberately bounded comparison set rather than as an exhaustive survey of image quality assessment measures. They represent four distinct and complementary approaches that are relevant to rendered-image evaluation: structural similarity (SSIM), learned feature-space similarity (LPIPS), combined structure–texture similarity (DISTS), and an explicit human-visual-system model of difference visibility and quality (HDR-VDP-2). SSIM and LPIPS are especially relevant to the reviewed contemporary rendering literature because they are frequently reported in neural-rendering benchmarks represented in the corpus, whereas HDR-VDP-2 and DISTS provide complementary perceptually motivated formulations. Metrics such as PSNR, FVD, and no-reference measures were therefore outside the predefined target-metric scope rather than assumed to be inferior. They are discussed where relevant to the interpretation of the included evidence, but a systematic comparison of all available image quality metrics was beyond the scope of the present review.

Rendering-specific research has long shown why this distinction matters. Čadík et al. demonstrated systematic weaknesses of objective image quality metrics when human observers evaluated computer-graphics artifacts [5]. Global-illumination methods have been compared with SSIM and HDR-VDP-2 [6], while game-engine studies directly compared metric-generated artifact maps with human markings of aliasing, Z-fighting, shadow acne, and peter-panning [7,8]. These studies showed that metric performance can depend on artifact morphology: SSIM performed strongly overall for spatially coherent errors, whereas HDR-VDP-2 could be more effective for some small or scattered differences [7,8]. Importantly, these early studies did not merely compare global scalar scores: several used spatially resolved predictions and observer-marked regions, making them unusually informative for understanding where a metric succeeds or fails within a rendered image. This distinction is relevant to later neural-rendering practice, where a single average score can conceal localized artifacts that dominate perceived quality.

Similar limitations arise for texture and material appearance. Griffin and Olano evaluated texture-compression error in final rendered images and showed that visibility is affected by interactions among texture content, mapping, gloss, bump information, and rendering conditions [9]. Azari et al. compared objective metrics with psychophysical judgments of rendered bidirectional texture functions and found that high structural similarity did not necessarily imply perceptual equivalence [10]. Studies of perception-oriented rendering approximation and artifact prediction further showed that visually acceptable simplifications and image-based rendering errors can be strongly content- and location-dependent [11,12].

The issue has become more prominent in the reviewed neural-rendering and novel-view-synthesis (NVS) literature, where SSIM and LPIPS were frequently reported beside conventional fidelity measures. HyperNeRF [13], GANeRF [14], S3IM [15], and ABLE-NeRF [16] illustrate this pattern within contemporary neural-field evaluation and optimization. Their frequent use within this literature improves benchmark comparability, but routine reporting does not by itself demonstrate agreement with human judgments. The rapid growth of this literature also affects how corpus-level frequencies should be interpreted: a metric may become common because it is embedded in benchmark conventions and public evaluation code rather than because it has been repeatedly validated for the perceptual construct under study. Separating adoption from validation is therefore central to this review.

Direct perceptual studies of neural rendering make this limitation explicit. Liang et al. found that commonly reported SSIM and LPIPS did not consistently provide the strongest correspondence with perceived NVS quality and could underperform PSNR under the tested conditions [17]. Onuoha et al. reported stronger performance for some learning-based measures, including DISTS, than for conventional SSIM-family measures [18]. Luo et al. compared SSIM, LPIPS, and DISTS with opinion scores for NeRF-generated scenes and again found content-dependent behavior [19], while metric-fusion work showed that no single measure was consistently optimal across all tested NeRF datasets [20]. The development of NeRF-specific no-reference assessment further reflects limitations of standard pixel-aligned full-reference comparisons for viewpoint-dependent synthesis artifacts [21].

More broadly, image similarity, image quality, fidelity, and perceived photorealism are related but distinct constructs. Image similarity describes correspondence between images according to a defined comparison rule; fidelity describes closeness to a chosen reference or intended target; image quality concerns the visual quality of the resulting image; and perceived photorealism concerns whether the rendered result is judged as a plausible photographic depiction. Improvement in one construct therefore does not necessarily imply improvement in the others. A rendering may achieve greater numerical similarity or reference fidelity without appearing more photorealistic, while a physically plausible change in lighting, material response, or surface detail may increase perceived realism despite reducing exact agreement with a reference image. In photorealistic avatars, SSIM and LPIPS were only weakly related to several subjective quality dimensions and were not designed specifically to predict realism [22]. HDR rendering similarly requires attention to luminance range and viewing conditions, with HDR-specific perceptual models outperforming generic alternatives on human-rated datasets [23]. Conversely, objective metrics can be useful when embedded in a constrained perceptual task: an SSIM-based foveated-denoising strategy, for example, was supported by a user study demonstrating comparable perceived quality at reduced computational cost [24]. The same caution applies to terminology. A method can improve image fidelity relative to a reference while leaving realism unchanged, or it can improve perceived realism through plausible material or lighting variation that is penalized by an exact-reference metric. For rendering research, the semantic meaning assigned to a score is therefore as important as the score itself.

These distinctions are especially relevant when controlled rendering attributes are manipulated. Lighting and global illumination have been assessed with SSIM, HDR-VDP-2, and more recently SSIM/LPIPS in relighting and dynamic-lighting systems [6,25]. Reflection and transmission are strongly viewpoint dependent, as illustrated by TR-Gaussians [26] and GlassGaussian [27], while material translucency can depend on subsurface light transport whose perceptual consequences are not necessarily represented by simple structural correspondence [28]. Contact-related shading requires similar care: ambient occlusion can support local grounding [29], but it should not be treated as equivalent to physically generated contact shadows, for which the observer-validated shadow-artifact studies provide more direct evidence [7,8]. Texture credibility likewise depends on how fine-scale material information survives rendering, mapping, compression, and viewing [9,10].

Against this background, the present review systematically examines HDR-VDP-2, LPIPS, DISTS, and SSIM in computer-generated and rendered imagery. It separates four questions that are often conflated in practice: what each metric is designed to measure; how it is used in rendering research; whether its output has been validated against human perception; and what evidence is available for controlled changes in lighting credibility, contact shadows, texture credibility, reflection credibility, surface micro-imperfections, and material translucency. The objective is not to identify a universal best metric, but to determine where each metric is informative and where its interpretation should remain limited.

Accordingly, the review addresses four research questions:

**RQ1.** What are the computational and perceptual principles underlying HDR-VDP-2, LPIPS, DISTS, and SSIM, and what types of image differences are they designed to quantify?

**RQ2.** How have HDR-VDP-2, LPIPS, DISTS, and SSIM been applied to the evaluation of computer-generated and rendered imagery?

**RQ3.** To what extent have the outputs of these metrics been validated against human judgments of perceptual quality, similarity, fidelity, or realism in rendering-related contexts?

**RQ4.** What evidence and limitations are relevant to the use of these metrics for controlled changes in lighting credibility, contact shadows, texture credibility, reflection credibility, surface micro-imperfections, and material translucency?

## 2. Materials and Methods

### 2.1. Review Design and Reporting Framework

This study was conducted as a systematic review with structured narrative synthesis of four objective full-reference image quality metrics—HDR-VDP-2, LPIPS, DISTS, and SSIM—in computer-generated and rendered imagery. Reporting followed PRISMA 2020 [30], and database-search reporting was additionally informed by PRISMA-S [31].

The review addressed the four research questions defined in the Introduction. A meta-analysis was not planned because the expected evidence was heterogeneous in rendering task, dataset, representation, metric implementation, human-evaluation design, and reported outcome. The eligibility criteria, coding rules, and synthesis framework were finalized before the evidence synthesis. The review was not prospectively registered. Following an editorial request during manuscript submission, the completed review was retrospectively registered on the Open Science Framework (OSF) on 2 September 2026 (https://doi.org/10.17605/OSF.IO/GWMNP). The registration documents the finalized review methods and is explicitly retrospective.

### 2.2. Eligibility Criteria

Original studies were eligible when at least one target metric was independently used to evaluate computer-generated or rendered visual output. Eligible contexts included traditional and real-time rendering, ray/path tracing, games, VR/AR, neural rendering, NeRF/NVS, 3D Gaussian splatting, avatars and digital humans, material/appearance modeling, synthetic rendering, simulation, and related reconstruction when a target metric assessed the rendered or synthesized output.

A target metric had to function as an output-evaluation measure, benchmark, or comparison metric. Studies were excluded when the metric appeared only as a training loss, internal optimization term, methodological citation, or incidental mention. Natural-image or photography studies without relevant rendered output were also excluded. When a paper used the same metric both during optimization and during final evaluation, it was retained only if an independently reported output-evaluation result could be identified. This rule was intended to prevent loss-function citations from inflating the application corpus.

Medical, dental, endoscopic, and related anatomical reconstruction studies were excluded when their primary purpose was clinical reconstruction rather than perceptual or image-level evaluation of rendered imagery. Secondary reviews without an original application or validation of a target metric were excluded, and reports that could not be retrieved in sufficient detail for report-level eligibility assessment were not included in the strict synthesis corpus.

For RQ1, the original publications defining SSIM, LPIPS, DISTS, and HDR-VDP-2 were additionally used as foundational methodological sources [1,2,3,4] but were not counted among the 283 application studies. Two foundational metric papers were encountered during database screening and were separated from the application corpus in the PRISMA accounting; the complete set of four original metric publications used for RQ1 was assembled from those screened records together with targeted known-item retrieval.

### 2.3. Information Sources

Systematic searches were conducted in Scopus, Web of Science Core Collection, and IEEE Xplore from database inception through 17 August 2026. Separate searches were run for HDR-VDP-2, DISTS, LPIPS, and SSIM, with no publication-year restriction. The three-source strategy was selected to combine broad multidisciplinary indexing across imaging and computer graphics through Scopus and Web of Science Core Collection with direct engineering and computing conference coverage through IEEE Xplore, while maintaining a consistent database set across the four metric-specific searches. ACM Digital Library was not included as a separate identification source. This was a scope decision rather than an assumption that ACM literature was unimportant: ACM publications could enter the corpus when indexed by the selected multidisciplinary databases, whereas ACM-only records remained outside the search frame. Consequently, the search may have underrepresented older conference-based computer-graphics and graphics-artifact studies, particularly work published primarily in ACM venues; this limitation is considered explicitly when interpreting the historical coverage of the review.

The searches yielded 523 records from Scopus, 280 from Web of Science, and 371 from IEEE Xplore, for 1174 records before deduplication. Metric-specific search yields and the exact database queries are provided in Supplementary Table S1.

Publisher, DOI, repository, exact-title, and institutional-authentication searches were subsequently used only to retrieve or verify reports already identified through the database searches; they did not constitute an additional identification stream. Database searching was completed on 17 August 2026, while renewed full-text retrieval and verification continued through 19 August 2026.

### 2.4. Search Strategy

Search strategies reflected the different specificity of the four metric names. HDR-VDP-2 and DISTS searches emphasized full names and recognized abbreviations, whereas LPIPS and SSIM were combined with broad rendering-context terms, including computer-generated or rendered imagery, 3D rendering, computer graphics, ray/path tracing, VR/AR, neural rendering, and novel-view synthesis, together with perceptual-evaluation terms such as image quality, similarity, realism, fidelity, and artifacts. The context vocabulary was intentionally based on broader rendering concepts rather than an exhaustive list of individual techniques, allowing emerging methods such as Gaussian splatting, avatar rendering, and reconstruction to be retrieved when described within these broader rendering contexts. The searches were not formally validated against a prespecified gold-standard set of known studies; the complete database-specific strategies are therefore provided in Supplementary Table S1 to make the scope and its limitations transparent.

Scopus searches used TITLE-ABS-KEY, Web of Science used TS, and IEEE Xplore queries were adapted to its search syntax. The complete reproducible strategies and search yields are preserved in Supplementary Table S1.

Search reporting followed PRISMA-S principles [31], including identification of information sources, final search date, database-specific strategies, and retrieved-record counts.

### 2.5. Record Management, Deduplication, and Study Selection

All database records were combined into one screening dataset. Duplicates were identified primarily through DOI matching and normalized-title comparison, with bibliographic quality-control checks. Of 1174 identified records, 494 duplicates were removed, leaving 680 unique records for title/abstract screening.

Title/abstract screening excluded 327 records. Two foundational metric papers encountered during screening were retained separately from the application corpus, leaving 351 reports sought for retrieval.

After the initial retrieval pass, 75 of the 351 reports sought remained unavailable. Renewed full-text retrieval through 19 August 2026 recovered 34 of these reports. Following full-text assessment, 33 of the recovered reports met the eligibility criteria and one was excluded because SSIM was mentioned only as a proposed future evaluation metric rather than being used for independent output evaluation. The final number of reports not retrieved was therefore 41: 40 appeared eligible from indexed evidence, while the target-metric role remained unverified in one report. The remaining unavailable reports were recorded as final PRISMA “reports not retrieved” outcomes rather than left as unresolved screening decisions. Retrieval attempts included institutional authentication, publisher and DOI links, repository searches, and exact-title searches. For the 40 reports that appeared potentially eligible from indexed evidence, the available metadata and abstracts did not provide sufficient methodological detail to determine reliably whether any contained an eligible subjective or human evaluation. None were therefore classified or counted as human-evidence in the strict corpus, although the possibility that some contained such evaluations cannot be excluded without the complete reports.

A total of 310 reports were assessed at report level. Twenty-seven were excluded after eligibility assessment and final extraction-stage protocol quality control, resulting in a strict application corpus of 283 studies with no open screening decisions.

Primary title/abstract screening and initial data extraction were performed by one reviewer. A second assessor subsequently conducted targeted independent verification rather than duplicating screening of the complete corpus. Verification focused on decisions with the greatest influence on the final synthesis: all 27 report-level exclusions, all 23 studies with human-evidence coding, all final RQ4 cue-evidence records, and the methodological-appraisal judgments for the 13 direct-validation studies. Any differences identified during verification were resolved by consensus before the final dataset was locked. Because the second assessor did not independently rescreen the complete set or a prespecified representative sample of the 680 title/abstract records, the verification procedure was designed as targeted error detection and classification quality control rather than as an inter-rater reliability study. Accordingly, disagreement frequencies were not quantified and no inter-rater agreement statistic was calculated. This approach provides secondary verification of the synthesis-critical decisions but does not eliminate the possibility of selection bias introduced during the single-reviewer primary screening.

### 2.6. Data Extraction

A standardized extraction framework recorded bibliographic information, target metric(s), rendering domain and application task, metric role, reference-comparison context, human-validation evidence, and the main evidence relevant to RQ1–RQ4. Non-target IQA measures reported as additional comparators were not systematically enumerated across all included studies; consequently, corpus-wide frequency counts for PSNR, FVD, or other non-target metrics were not calculated.

Metrics were coded only when independently reported as output-evaluation measures. Human evidence was extracted when observers judged quality, similarity, fidelity, realism, artifact visibility, or another perceptual construct relevant to the target metric. Where available, participant numbers, tasks, MOS, correlations, ranking agreement, ROC/AUC, and other metric–human relationships were recorded.

Six additional fields captured evidence related to lighting credibility, contact shadows, texture credibility, reflection credibility, surface micro-imperfections, and material translucency. Cue coding was based on the role of the visual phenomenon in the study rather than keyword occurrence alone.

### 2.7. RQ-Specific Coding

For RQ1, the four metrics were compared according to computational representation, perceptual basis, treatment of structure/texture, luminance dependence, output interpretation, and conceptual limitations, using the foundational sources [1,2,3,4].

For RQ2, each study was assigned one mutually exclusive primary application family: neural rendering, NVS, and 3D reconstruction; material, appearance, and lighting; IQA, artifact, and perceptual assessment; avatar and digital-human rendering; traditional rendering, enhancement, and optimization; VR, display, and immersive rendering; or synthetic data, simulation, and application-specific rendering. The primary family was assigned according to the study’s main stated objective and the principal rendering or evaluation task for which the target metric was reported. Secondary techniques, datasets, or application contexts did not generate additional RQ2 assignments. When more than one category was plausible, the category most closely corresponding to the study’s primary contribution and evaluated output was selected. RQ2 classification was therefore mutually exclusive for descriptive counting, whereas metric and RQ4 cue coding remained non-exclusive.

For RQ3, human evidence was graded as direct metric–human validation only when target-metric outputs were explicitly linked to human judgments through quantitative, spatial, psychophysical, ranking, correlation, classification, or equivalent analyses. The defining threshold was therefore an explicit analysis of the relationship between metric output and human ground truth; merely reporting an objective metric and a subjective evaluation within the same study was not sufficient. Studies containing both forms of evaluation but not directly testing their relationship were coded as parallel/indirect evidence.

For RQ4, the six rendering attributes were defined a priori as the review’s applicability framework before the final evidence synthesis; they were not derived from frequency patterns in the included corpus. The framework originated from a previously published taxonomy of photorealism cues in 3D-rendered architectural imagery, but the six attributes were used here because they describe physically and perceptually interpretable properties of rendered appearance that can also occur across broader rendering domains. They were not intended as an exhaustive taxonomy of all factors affecting photorealism, and other attributes outside these predefined six cues were not systematically coded. Evidence was graded as Direct, Supportive, or Adjacent. Direct evidence required the cue itself to be an explicit component of the rendering method, manipulation, artifact, or evaluation. Supportive evidence was assigned when the evaluated phenomenon was not identical to the target cue but shared the same underlying appearance mechanism or perceptual consequence closely enough to inform interpretation of that cue. Adjacent evidence was assigned when a phenomenon was conceptually related to the cue but differed in its physical origin, image formation, or perceptual interpretation and therefore could not be treated as evidence that the metric measured the target cue itself. Contextual-only and false keyword matches were removed from final cue counts. For example, ambient occlusion was classified as Adjacent rather than Supportive evidence for contact shadows. Although both can increase local darkening and visual grounding near geometric contact, ambient occlusion is an approximation of occluded ambient illumination and is not equivalent to a cast or contact shadow generated by direct-light visibility. It was therefore considered conceptually relevant to contact perception but insufficiently equivalent in image formation and physical mechanism to support claims about metric sensitivity to contact shadows themselves. Likewise, participating-media scattering was not treated as material translucency unless the study explicitly concerned translucent material appearance. This conservative coding was intended to avoid overstating the amount of cue-specific evidence. Importantly, the presence of a target metric in a study involving one of these attributes was treated as evidence of application to that cue, not as evidence that the metric perceptually measures the cue well. Claims about perceptual validity required separate human-linked evidence, and direct metric–human validation required an explicit analysis relating metric output to observer judgments.

### 2.8. Methodological Quality and Evidence Appraisal

Because the corpus combined heterogeneous computational, rendering, IQA, VR, neural-rendering, and observer-study designs, no single validated clinical-style risk-of-bias instrument was considered appropriate across all 283 application studies. The descriptive RQ2 synthesis therefore required verified independent use of a target metric as an output-evaluation measure, while RQ4 confidence was handled through explicit Direct, Supportive, and Adjacent evidence grades.

For RQ3, where the review makes inferential claims about agreement between objective metrics and human perception, the 13 studies classified as direct metric–human validation underwent an additional full-text methodological appraisal. The appraisal was a review-specific structured framework rather than a previously validated risk-of-bias instrument. It was developed to capture methodological features that directly affect interpretation of metric–human agreement across the heterogeneous observer-study and computer-graphics designs represented in the corpus. Six domains were assessed: (1) clarity and appropriateness of the perceptual construct or observer task; (2) reporting of the human sample or source subjective dataset; (3) adequacy and description of stimulus/content coverage; (4) reporting and control of viewing or presentation conditions, where applicable; (5) transparency of target-metric and reference-comparison implementation; and (6) appropriateness of the statistical, spatial, psychophysical, or ranking analysis linking metric output to human ground truth.

Each domain was judged as Low concern, Some concerns, High concern, or Not applicable. The overall judgment followed an explicit rule: Low concern required all applicable domains to be rated Low concern; Some concerns was assigned when at least one applicable domain was rated Some concerns and none was rated High concern; High concern was reserved for one or more High-concern domains judged material to interpretation. Not-applicable domains were ignored when deriving the overall judgment. No numerical quality score was calculated. The ten studies containing parallel/indirect human evidence were not appraised as direct validation because they did not test a target metric against human ground truth. The methodological-appraisal judgments for all 13 direct-validation studies were independently verified by the second assessor, and any differences in domain-level or overall judgments were resolved by consensus. Study-level judgments and supporting notes are provided in Supplementary Table S3.

This appraisal was intentionally restricted to the direct-validation subset because these were the studies from which the review draws conclusions about agreement between objective metrics and human perception. Applying the same framework to studies that merely reported routine metric use, without a metric–human comparison, would not have addressed the same evidential question. The appraisal is therefore intended to characterize confidence in the human-validation evidence rather than to imply a common intervention-style risk of bias across heterogeneous computer-graphics studies. Evidence availability, directness, methodological concern, and applicability to the review questions were kept conceptually separate.

### 2.9. Data Synthesis, Statistical Analysis, and Availability Sensitivity Analysis

Evidence was synthesized using descriptive statistics and structured narrative synthesis. Counts and percentages refer to the strict corpus of 283 studies. Target-metric frequencies were non-exclusive because studies could report multiple metrics, whereas RQ2 primary application families were mutually exclusive.

For RQ3, studies with any relevant human evidence were counted first and then separated into direct metric–human validation and parallel/indirect evidence; metric-specific validation counts were non-exclusive.

For RQ4, evidence was counted separately for each cue and grade. Cue assignments were non-exclusive because one study could inform more than one rendering attribute, while contextual-only matches were excluded.

Meta-analysis was not performed because studies differed substantially in rendered content, algorithms, datasets, metric implementations, score scales, reference definitions, perceptual constructs, observer tasks, and statistical relationships. Raw metric values were therefore not normalized or pooled across studies, and absolute SSIM, LPIPS, DISTS, or HDR-VDP-2 scores from traditional graphics and neural-rendering benchmarks were not treated as directly comparable. Cross-domain comparisons were instead performed at the level of study counts, application families, evidence categories, and within-study metric–human relationships, with metric findings interpreted within the reference and evaluation context defined by each study. The synthesis therefore emphasizes application prevalence, strength of human validation, context-dependent agreement, and cue-specific evidence gaps.

To examine whether the 40 reports that still appeared eligible from indexed evidence but could not be retrieved might materially alter the principal prevalence findings, an availability sensitivity analysis was performed using indexed metadata only. This analysis did not reclassify the reports as included studies. Instead, it calculated an extreme-case augmented corpus in which all 40 apparently eligible reports were assumed to satisfy full-text eligibility. The remaining unretrieved report, for which the target-metric role could not be verified, was excluded from this sensitivity analysis because independent output-evaluation use could not be established.

No formal assessment of publication/reporting bias or a single overall certainty-of-evidence grade was performed because no common effect estimate or homogeneous outcome was synthesized across the heterogeneous rendering, IQA, neural-rendering, VR, and observer-study designs. Confidence in RQ3 was instead characterized through study-level methodological appraisal and evidence directness, while RQ4 confidence was represented through the Direct, Supportive, and Adjacent evidence grades. Potential availability bias arising from unavailable reports was examined separately through the sensitivity analysis described above.

### 2.10. Use of Generative AI Tools

OpenAI ChatGPT using GPT-5.6 Sol (OpenAI; accessed August 2026) was used as an assistive tool for data organization, preliminary coding support, quality-control checks, calculation verification, manuscript drafting, and language editing. AI-assisted outputs were not treated as source evidence. Final eligibility decisions, extracted evidence, quantitative summaries, methodological-appraisal judgments, cue classifications, and interpretations were manually verified against the underlying reports or indexed source records by M.G. and, where applicable, independently checked by A.B. as described above. The authors reviewed and edited all AI-assisted output and take full responsibility for the final content.

## 3. Results

### 3.1. Study Selection

The searches identified 1174 records. After removal of 494 duplicates, 680 unique records were screened; 327 were excluded at the title/abstract stage and two foundational metric papers were retained separately, leaving 351 reports sought for retrieval.

Forty-one reports could not be retrieved in sufficient detail for report-level assessment (40 appeared potentially eligible based on indexed evidence, and one had an unverified target-metric role). These reports were recorded as final PRISMA retrieval outcomes and excluded from the strict synthesis corpus. Renewed retrieval recovered 34 reports that had initially been unavailable; 33 were subsequently included, and one was excluded after full-text assessment.

**Figure 1:**
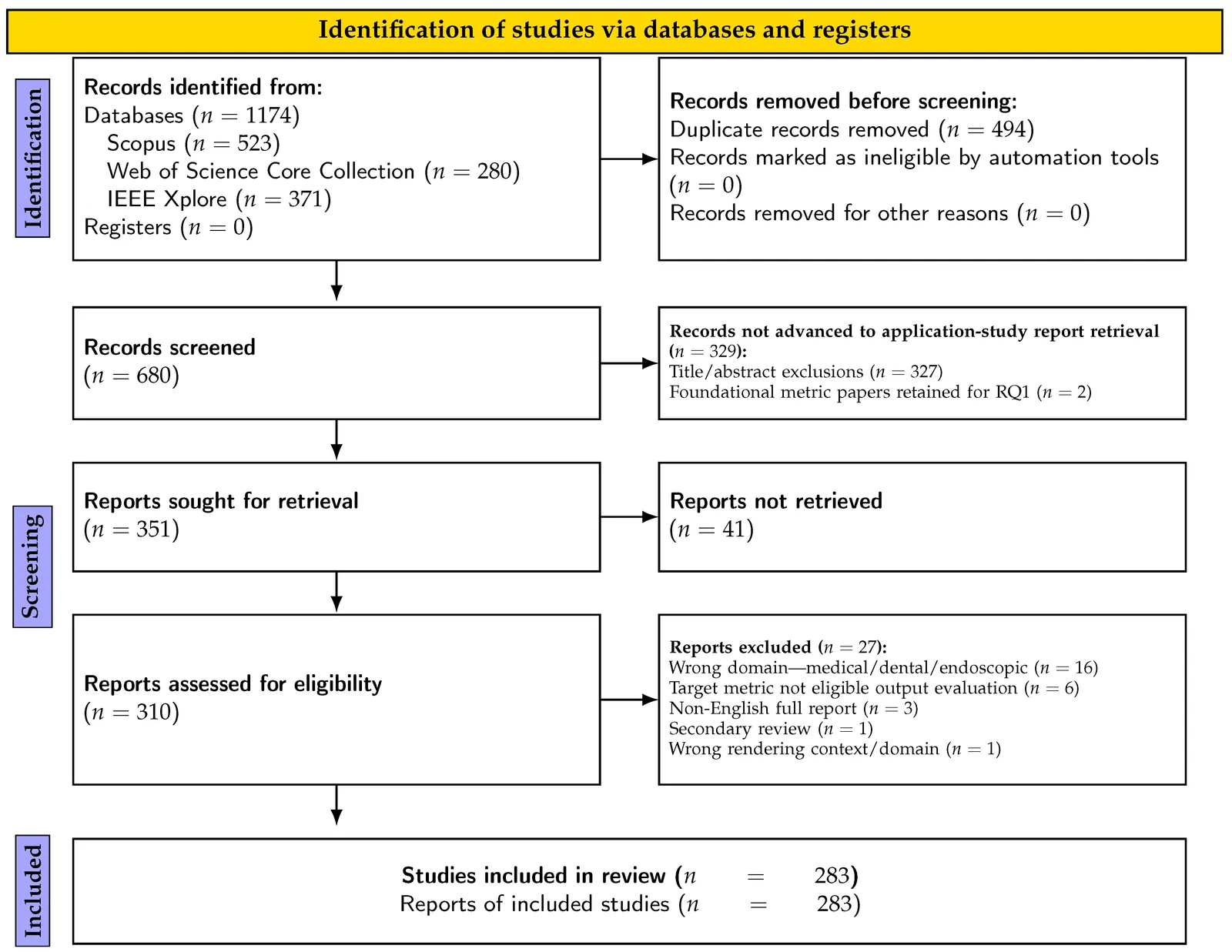
PRISMA 2020 flow diagram of study identification, screening, retrieval, eligibility assessment, and inclusion. Note: Database-specific record counts are reported within the identification box. Of the 329 records not advanced to application-study report retrieval, 327 were excluded at title/abstract screening and two foundational metric papers were retained separately for the RQ1 conceptual synthesis; these two papers were not counted in the application corpus. Source: Page et al. [30]; adapted from the PRISMA 2020 flow-diagram template (CC BY 4.0).

The remaining 310 reports were assessed for eligibility, of which 27 were excluded at report level, including final extraction-stage protocol corrections and the one newly recovered report in which SSIM was only proposed for future work. The strict application corpus therefore comprised 283 studies with no unresolved screening decisions. Figure 1 summarizes the PRISMA 2020 flow [30].

The availability sensitivity analysis did not change the principal prevalence pattern. Among the 40 apparently eligible but still not-retrieved reports, indexed metadata identified SSIM in 13 and LPIPS in 33 reports (non-exclusive). Under the deliberately conservative assumption that all 40 were eligible, the augmented corpus would contain 323 studies, with SSIM in 241 (74.6%) and LPIPS in 180 (55.7%); SSIM would therefore remain the most prevalent target metric and LPIPS the second. Thirty-two of the 40 apparently eligible reports concerned neural rendering or novel-view synthesis, which would increase that application family from 175/283 (61.8%) to 207/323 (64.1%) rather than changing the corpus-level conclusion that recent neural rendering dominates the evidence base. This sensitivity analysis addresses availability bias only and cannot determine whether human-validation findings would change if the remaining full texts were obtained.

### 3.2. Characteristics of the Included Studies

The 283 included studies spanned 2010–2026 but were strongly concentrated in recent work: 14 (4.9%) were published in 2010–2020, 30 (10.6%) in 2021–2023, and 239 (84.5%) in 2024–2026. Table 1 summarizes the overall characteristics of the strict application corpus.

SSIM appeared in 228 studies (80.6%) and LPIPS in 147 (51.9%), whereas HDR-VDP-2 and DISTS each appeared in four (1.4%). The most common configurations were SSIM only (130; 45.9%), SSIM + LPIPS (91; 32.2%), and LPIPS only (54; 19.1%). Metric frequencies are non-exclusive.

Earlier studies focused more often on conventional graphics artifacts, global illumination, material/texture representation, and rendering approximation [5,6,7,8,9,10,11,12]. The recent corpus was dominated by neural scene representations and NVS, including dynamic neural fields [13], adversarially optimized NeRFs [14], synthetic-to-real NeRFs [32], and comparisons of contemporary neural-rendering representations [33].

### 3.3. RQ1: Computational and Perceptual Principles of the Target Metrics

The four target metrics differed in both representation and perceptual interpretation. Table 2 compares their computational principles, perceptual constructs, and principal limitations for evaluating rendering realism.

SSIM compares local luminance, contrast, and structure and is best interpreted as a structural-similarity measure rather than an explicit model of visual visibility or realism [2].

LPIPS measures distance in learned deep feature space and targets perceptual similarity beyond independent pixel differences [3]. DISTS combines deep structural correlations with texture statistics, allowing some texture variation to be treated differently from structural change [4].

HDR-VDP-2 explicitly models luminance-dependent visual sensitivity and masking to predict the visibility and quality impact of image differences [1].

Accordingly, the four metrics quantify complementary rather than identical properties: structure (SSIM), learned feature similarity (LPIPS), structure-texture similarity (DISTS), and HVS-based difference visibility/quality (HDR-VDP-2).

RQ1 therefore does not support treating the four scores as interchangeable numerical proxies for photorealism; each metric should be interpreted according to the perceptual construct it was designed to represent. Interpretation also depends on the reference image against which the evaluated rendering is compared. Across the corpus, common reference contexts included held-out or captured ground-truth views in neural rendering, novel-view synthesis, and reconstruction; higher-quality, less-degraded, or original outputs in traditional rendering, denoising, compression, and enhancement tasks; and captured, measured, or high-quality reference appearances in some material-focused applications. Reference definitions were too heterogeneous for meaningful quantitative pooling. Importantly, similarity to any of these references indicates fidelity to the selected target and should not automatically be interpreted as perceived quality or photorealism.

### 3.4. RQ2: Applications in Computer-Generated and Rendered Imagery

Neural rendering/NVS/3D reconstruction was the largest application family (175/283; 61.8%), followed by material/appearance/lighting (26; 9.2%), IQA/artifact and perceptual assessment (21; 7.4%), traditional rendering/enhancement/optimization (20; 7.1%), avatar/digital-human rendering (19; 6.7%), VR/display/immersive rendering (17; 6.0%), and synthetic-data/simulation/application-specific rendering (5; 1.8%).

SSIM occurred across all seven families and functioned mainly as a general-purpose fidelity benchmark. LPIPS was strongly associated with recent neural rendering and NVS, where it was frequently reported with SSIM and PSNR [13,14,15,16,32,33,34]. At the study level, 139 of the 175 neural-rendering/NVS/3D-reconstruction studies used SSIM and 107 used LPIPS. In the material/appearance/lighting family, SSIM appeared in 20 of 26 studies and LPIPS in 12. By contrast, all four DISTS applications and three of four HDR-VDP-2 applications remained concentrated in the IQA/artifact-perception family, emphasizing their comparatively specialized use. Table 3 summarizes target-metric use across the seven application families.

HDR-VDP-2 and DISTS were far less common and concentrated in explicitly perceptual/IQA-oriented work. HDR-VDP-2 appeared in global-illumination and graphics-artifact studies [6,7,8], whereas all DISTS applications were located within perceptual-assessment contexts.

Thus, RQ2 shows that metric choice is shaped strongly by application convention within the reviewed corpus: among the four target metrics, SSIM was the most prevalent, LPIPS was particularly characteristic of learned-rendering studies, and HDR-VDP-2 and DISTS remained comparatively specialized rather than routine benchmarks.

### 3.5. RQ3: Validation Against Human Judgments

Only 23 of 283 studies (8.1%) contained human or subjective evidence relevant to quality, similarity, fidelity, realism, or artifact perception. Thirteen studies (4.6%) directly compared target-metric behavior with human ground truth, while ten (3.5%) reported objective and subjective evaluation in parallel without directly testing their relationship.

Direct validation involved SSIM in 12 studies, LPIPS in 3, DISTS in 4, and HDR-VDP-2 in 3; these counts overlap because individual studies could evaluate multiple metrics. The contrast with overall adoption was substantial: routine benchmark use was common, whereas formal perceptual validation was rare. Relative to their overall use, direct human-validation evidence was available for only a small fraction of SSIM and LPIPS applications, whereas the much smaller DISTS and HDR-VDP-2 application sets were proportionally more represented in perceptual-validation studies. These ratios should not be interpreted as performance rankings, but they highlight different evidence profiles. The direct-validation set also spanned non-aligned NVS, compressed avatar animation, and BTF material appearance [35,36,37].

**Figure 2:**
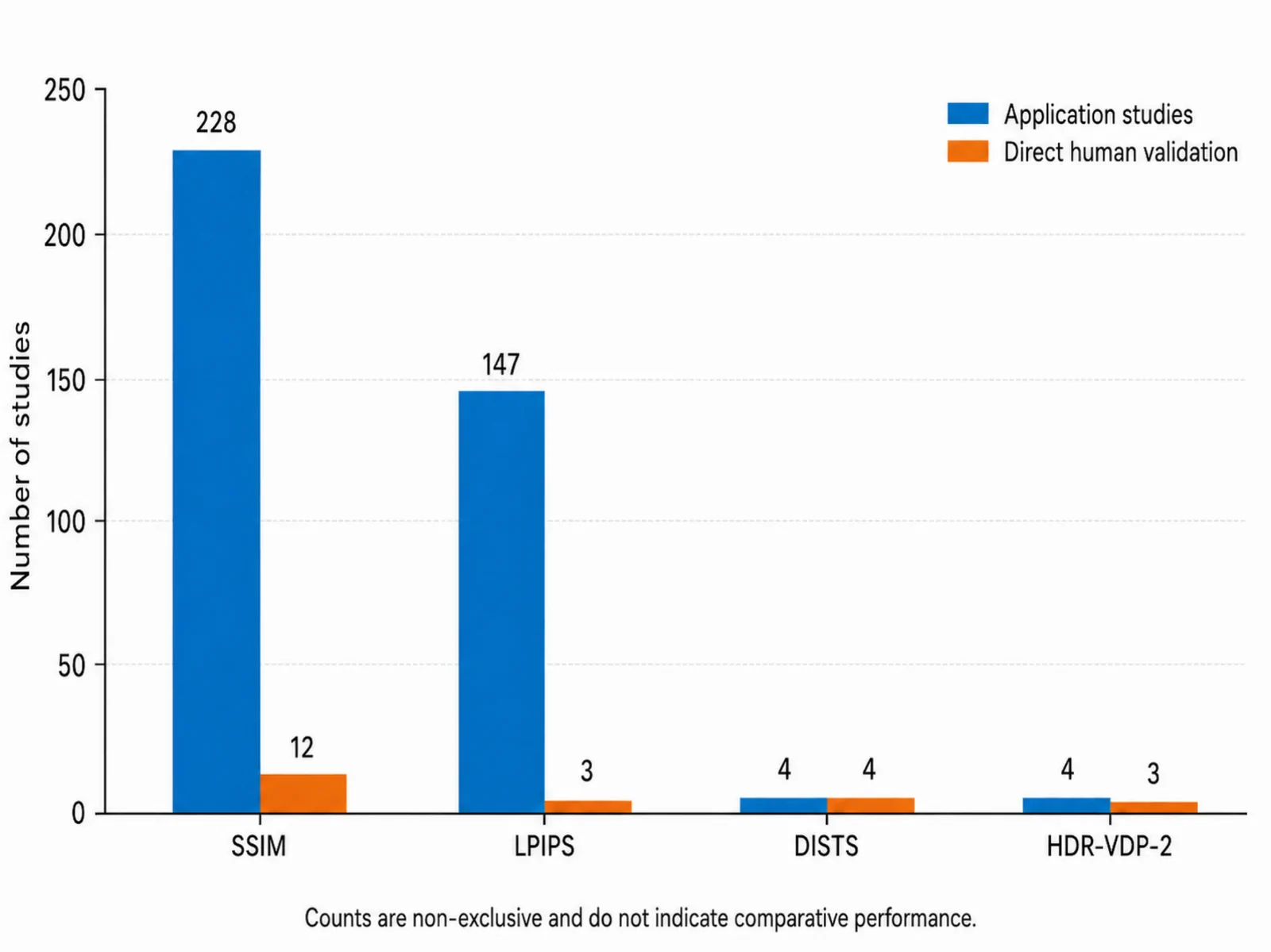
Application frequency and direct metric–human validation evidence for the four target metrics. Counts are non-exclusive because individual studies could report more than one metric. Validation counts indicate the amount of direct human-linked evidence identified for each metric and should not be interpreted as comparative performance rankings.

Figure 2 contrasts the overall adoption of the four target metrics with the amount of direct metric–human validation evidence identified for each metric. This comparison should be interpreted as an evidence-coverage analysis rather than as a comparison of metric performance. “Adoption” denotes the number of application studies in which a metric was independently reported for output evaluation, whereas “direct validation” denotes studies that explicitly tested the relationship between that metric and human judgments. A larger validation count therefore indicates that a metric has been tested against human evidence more often, not that it necessarily agreed better with observers. Conversely, high adoption with little direct validation indicates widespread benchmark use without a correspondingly large perceptual-validation literature.

#### 3.5.1. Graphics Artifacts and Spatially Localized Rendering Errors

Graphics-artifact studies provided some of the most direct rendering-specific validation. Čadík et al. compared localized metric predictions with judgments from 35 observers and identified failures for brightness/contrast changes, near-threshold differences, and higher-level illumination plausibility [5].

Piórkowski and Mantiuk compared SSIM/HDR-VDP-2 maps with observer markings of aliasing, Z-fighting, shadow acne, and peter-panning [7], and an extended 55-observer study replicated the main pattern [8]. SSIM showed very high overall ROC/AUC agreement, whereas HDR-VDP-2 was particularly competitive for small or scattered aliasing, demonstrating artifact-dependent rather than universal metric performance. In the first study, overall AUC was approximately 0.973 for SSIM and 0.957 for HDR-VDP-2, while the extended study again found SSIM most consistent overall and HDR-VDP-2 particularly strong for aliasing. The important result is therefore not the small numerical difference between metrics, but the change in relative behavior across artifact types.

These studies show that the spatial form of a rendering error can influence which objective measure best corresponds with observer responses.

#### 3.5.2. Neural Rendering and Novel-View Synthesis

Neural-rendering validation likewise produced no universal ranking. Liang et al. used pairwise judgments from 39 participants and found PSNR significantly better than SSIM and LPIPS for the tested NVS quality ranking, while DISTS was among the evaluated perceptual measures [17].

Onuoha et al. found DISTS among the stronger correlates of subjective NeRF quality [18], and Luo et al. confirmed with MOS from 86 participants that SSIM, LPIPS, and DISTS differed across synthetic and realistic content, with DISTS among the stronger full-reference metrics [19]. These findings are especially relevant because NVS errors are not limited to uniform noise or blur: local geometry mistakes, view-dependent appearance failures, temporal inconsistency, and disocclusion artifacts can influence subjective judgments differently. A single full-reference score can therefore summarize several perceptually distinct failure modes.

Metric-fusion experiments further showed that no single measure was consistently optimal across all NeRF datasets [20]. Non-aligned reference work based on expert preferences also showed that geometric misalignment can undermine conventional full-reference comparisons in NVS [35].

Overall, NVS metric–human agreement depended on scene content, alignment, artifact type, temporal presentation, and the perceptual task.

#### 3.5.3. Material Appearance and Texture

Material studies similarly showed that high structural similarity does not guarantee perceptual equivalence. In rendered BTF comparisons, SSIM values remained very high across tested resolution pairs even when human discriminability differed substantially [10].

BTF compression work combined SSIM with psychophysical material judgments [37], while rendered texture-compression experiments showed that visibility depends on interactions among texture, mapping, geometry, gloss, bump information, and lighting [9].

#### 3.5.4. Avatars, Realism, and Multidimensional Quality

Avatar studies highlighted the distinction between similarity and realism. In multidimensional photorealistic-avatar evaluation, SSIM and LPIPS correlated weakly with perceived realism but more strongly with selected attributes such as emotion accuracy [22].

For compressed facial animation, objective-metric correlations with subjective MOS were only moderate and alternative measures could perform better [36], reinforcing that a metric may align with one perceptual construct but not another.

#### 3.5.5. Parallel Subjective Evidence

Ten studies were retained as parallel human evidence because subjective and objective evaluations were reported without direct metric–human correlation. Examples included VR reflection rendering [38], expert assessment of reconstructed museum artifacts [39], VR panorama synthesis [40], and foveated ray-tracing validation [24]. A newly recovered VR-training study likewise reported SSIM alongside post-session Likert ratings from 50 participants for the same system comparisons, but did not test a direct association between the objective and subjective outcomes; it was therefore retained as parallel rather than direct validation (Supplementary Table S3).

Keeping these studies separate prevented convergent objective and subjective outcomes from being interpreted automatically as formal validation of the target metric.

RQ3 therefore supports a context-dependent interpretation: no target metric consistently reproduced human judgments across rendering domains, artifacts, contents, and perceptual constructs. Table 4 summarizes representative direct human-validation evidence.

#### 3.5.6. Methodological Appraisal of Direct Validation Studies

The domain-based appraisal of the 13 direct metric–human validation studies identified seven studies with an overall Low concern judgment and six with Some concerns; no study was assigned an overall High concern judgment. The strongest studies typically combined a clearly defined perceptual task with controlled or well-documented presentation conditions and an explicit statistical or spatial comparison between metric output and human ground truth. Examples included controlled graphics-artifact experiments [5,7,8], calibrated NVS testing [17], and standardized avatar-quality assessment [36].

The most common sources of concern were not evidence of invalid results, but limitations in generalizability or reporting: reliance on pre-existing subjective datasets whose original viewing conditions were inherited rather than re-established [19,20,23], online/crowdsourced presentation with incomplete control of the display environment [18], a small expert panel in non-aligned NVS assessment [35], or less complete reporting of viewing/statistical details in older psychophysical material studies [37]. The complete domain-level judgments are reported in Supplementary Table S3 and were considered when interpreting RQ3.

### 3.6. RQ4: Evidence Relevant to Six Rendering Attributes

RQ4 evidence was highly uneven. Final coding identified 19 direct lighting studies, 2 direct contact-shadow studies, 7 direct and 4 supportive texture studies, 8 direct and 1 supportive reflection studies, 1 direct and 2 supportive micro-imperfection studies, and 2 direct translucency studies. Ambient occlusion contributed one adjacent contact-shadow record rather than direct evidence. Table 5 summarizes the evidence mapped to the six rendering attributes.

**Figure 3:**
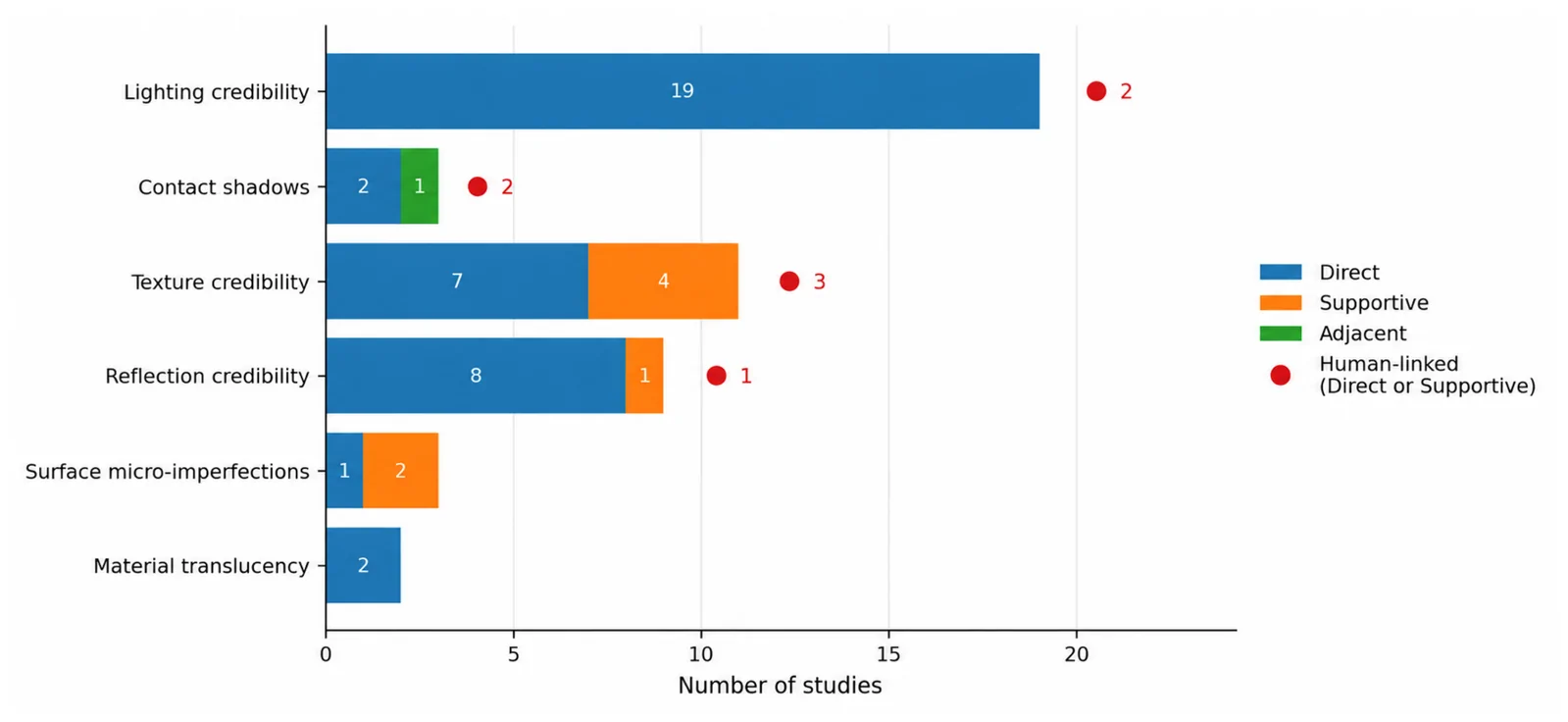
Distribution and evidential strength of studies across the six predefined rendering attributes. Bars show the number of records classified as Direct, Supportive, or Adjacent under the cue-specific evidence-grading framework (Section 2.7). Red markers denote human-linked records within the Direct/Supportive evidence; these counts are subsets and are not additive.

Figure 3 provides a visual summary of the distribution and evidential strength of studies across the six predefined rendering attributes.

The evidence categories shown in Figure 3 describe relevance to each rendering attribute and should not be confused with the RQ3 classification of metric–human validation. In the RQ4 framework, Direct, Supportive, and Adjacent indicate the strength and proximity of evidence to a specific rendering cue. The human-linked count identifies records classified as Direct or Supportive for that cue that also contained relevant human or subjective evidence. A human-linked record was not necessarily a direct metric–human validation study: the latter classification additionally required an explicit analysis linking target-metric output to human judgments, as defined for RQ3. Adjacent records were reported separately and were not included in the human-linked Direct/Supportive count.

#### 3.6.1. Lighting Credibility

Lighting had the broadest application base, covering global illumination, relighting, dynamic illumination, low-light reconstruction, and HDR rendering. Relevant examples ranged from traditional GI comparison [6] to dynamic-lighting Gaussian splatting [25], Ambient-NeRF [41], SAwareSSGI [42], CycleGAN GI estimation [43], Neural Scene Baking [44], URHand [45], and GroomLight [46]. The direct application set included both classical illumination problems and recent learned representations, so the cue count reflects breadth of engineering use rather than one standardized lighting experiment. This explains why application volume was much larger than the human-linked evidence count.

Despite 19 direct applications, only two lighting-related records were linked to relevant human evidence. The literature therefore supports widespread use of objective metrics under changing illumination more strongly than it supports their interpretation as direct measures of perceived lighting credibility.

#### 3.6.2. Contact Shadows

Contact-shadow evidence was sparse but methodologically strong. The two direct studies used observer annotations of shadow acne and peter-panning and compared them with SSIM/HDR-VDP-2 predictions [7,8]. AOGAN was retained as adjacent evidence because ambient occlusion can contribute to local grounding but differs from cast/contact shadows in both its underlying illumination mechanism and image formation; it was therefore considered conceptually relevant without being treated as evidence that the evaluated metric measured contact shadows themselves [29].

#### 3.6.3. Texture Credibility

Texture credibility had seven direct and four supportive studies. Human-grounded evidence came primarily from BTF/material experiments [10,37] and rendered texture-compression work [9]. Recent learned-rendering applications included Frequency-Modulated Point Cloud Rendering [47], PointDreamer [48], texture-consistent NeRF style transfer [49], and expert-supported virtual-puppetry reconstruction [39]. Three texture-related records were linked to human or expert evidence, making texture one of the better supported material cues in the review, although the evidence still spans substantially different constructs such as resolution discrimination, compressed BTF appearance, and expert assessment of reconstructed artifacts.

The combined evidence indicates that texture fidelity is context dependent and can interact with material, mapping, lighting, geometry, and viewing conditions; structural similarity alone may therefore miss some perceptually relevant material changes.

#### 3.6.4. Reflection Credibility

Reflection credibility was represented by eight direct and one supportive study. The VR reflection study combined SSIM with subjective realism ratings [38], while ABLE-NeRF [16], TR-Gaussians [26], multi-bounce NeRF ray tracing [50], and GlassGaussian [27] addressed glossy, reflective, transparent, or view-dependent appearance. Newly recovered full texts added direct evidence from 3DGS-based reflection baking, spatially varying BRDF estimation, and an SSIM comparison of game-engine and offline PBR outputs. The VR reflection experiment remained the clearest human-related case because the hybrid reflection approach improved both objective SSIM and subjective realism ratings, yet the absence of a reported direct SSIM-rating correlation means it remains convergent rather than formal metric validation.

These applications demonstrate routine use of full-reference metrics for reflective rendering, but direct metric–human validation of reflection credibility remains very limited.

#### 3.6.5. Surface Micro-Imperfections

Surface micro-imperfections remained a clear evidence gap. GlassGaussian was the only direct example because non-ideal glass-surface imperfections were central to the method [27]. GroomLight [46] and one newly recovered spatially varying BRDF study provided supportive roughness-related evidence, but neither independently manipulated scratches, wear, smudges, or comparable stochastic surface micro-imperfections. No direct metric–human validation of micro-imperfection realism was identified.

#### 3.6.6. Material Translucency

Material translucency was similarly sparse. A biophysical skin model explicitly represented layered tissue and subsurface light transport [28], while ABLE-NeRF provided a second application involving transparent/translucent appearance [16]. Transparency, participating-media scattering, and subsurface material translucency were kept conceptually distinct during coding.

No direct human-validation study established how SSIM, LPIPS, DISTS, or HDR-VDP-2 responds to perceptually meaningful changes in subsurface scattering or material translucency.

#### 3.6.7. Overall RQ4 Pattern

Across the six cues, lighting had the broadest application coverage; contact shadows had little volume but unusually strong observer validation; texture and reflection were moderately represented; and micro-imperfections and translucency remained the clearest evidence gaps. The six attributes correspond to the previously published cue-based taxonomy used as the predefined RQ4 applicability framework [51].

All 283 included application studies are individually identified in Supplementary Table S2 with their manuscript reference numbers. The included studies not otherwise cited individually in the narrative are cited collectively here [52,53,54,55,56,57,58,59,60,61,62,63,64,65,66,67,68,69,70,71,72,73,74,75,76,77,78,79,80,81,82,83,84,85,86,87,88,89,90,91,92,93,94,95,96,97,98,99,100,101,102,103,104,105,106,107,108,109,110,111,112,113,114,115,116,117,118,119,120,121,122,123,124,125,126,127,128,129,130,131,132,133,134,135,136,137,138,139,140,141,142,143,144,145,146,147,148,149,150,151,152,153,154,155,156,157,158,159,160,161,162,163,164,165,166,167,168,169,170,171,172,173,174,175,176,177,178,179,180,181,182,183,184,185,186,187,188,189,190,191,192,193,194,195,196,197,198,199,200,201,202,203,204,205,206,207,208,209,210,211,212,213,214,215,216,217,218,219,220,221,222,223,224,225,226,227,228,229,230,231,232,233,234,235,236,237,238,239,240,241,242,243,244,245,246,247,248,249,250,251,252,253,254,255,256,257,258,259,260,261,262,263,264,265,266,267,268,269,270,271,272,273,274,275,276,277,278,279,280,281,282,283,284,285,286,287,288,289,290].

The evidence does not justify assigning one universal metric to each cue. Instead, metric choice should reflect the specific rendering difference, perceptual construct, and strength of available validation evidence.

## 4. Discussion

### 4.1. Principal Findings

Three main findings emerge from this review. First, SSIM, LPIPS, DISTS, and HDR-VDP-2 are not interchangeable: they quantify structural similarity, learned feature distance, combined structure–texture similarity, and HVS-based visibility/quality, respectively [1,2,3,4]. Second, metric adoption and perceptual validation are markedly imbalanced: among the four target metrics in the reviewed corpus, SSIM and LPIPS were by far the most frequently reported, yet only 23 of 283 studies contained relevant human evidence and only 13 directly compared target-metric outputs with human judgments. Third, cue-specific evidence is uneven, with broad coverage for lighting, sparse but direct observer validation for contact shadows, moderate evidence for texture and reflection, and major gaps for micro-imperfections and translucency.

The main implication is that frequent metric use should not be interpreted as evidence of perceptual validity. SSIM and LPIPS are convenient and widely implemented, particularly in the 175-study neural-rendering/NVS/3D-reconstruction family, but repeated benchmark use does not demonstrate that score differences correspond to perceived quality or realism. Conversely, DISTS and HDR-VDP-2 were uncommon but proportionally more often represented in studies with explicit perceptual validation. This contrast reflects different patterns of adoption and validation, not evidence that the less frequently used metrics are universally superior.

The relevant question is therefore not which metric is universally best, but which visual property or perceptual construct is being evaluated and whether the chosen metric has been shown to respond meaningfully to that specific task.

### 4.2. Metric Prevalence Should Not Be Interpreted as Perceptual Validity

Combinations of PSNR, SSIM, and LPIPS were frequently used in the neural-rendering studies represented in the reviewed literature [13,14,15,16,32,33,34], providing a common benchmark language within this body of work. However, human-validation studies show that this convention can become misleading when numerical fidelity is treated as a proxy for human perception. SSIM and LPIPS underperformed PSNR in one controlled NVS study [17]; DISTS performed strongly in other NeRF datasets [18,19]; and no metric was consistently optimal across datasets [20]. Avatar realism produced similarly weak SSIM/LPIPS associations for several subjective dimensions [22]. This distinction should be made explicit whenever rendering studies move from statements such as “method A achieved a better SSIM/LPIPS score” to perceptual claims such as “method A looks more realistic”. Claims should therefore distinguish statistical or numerical improvement from perceptually demonstrated improvement.

These results support a distinction between benchmark adoption and construct validity. A metric can be useful for reproducible engineering comparison without being a validated predictor of realism, quality, or preference. Accordingly, metric outputs should be interpreted at the level of the construct they actually quantify. Greater structural agreement, lower learned-feature distance, or greater reference fidelity may coincide with improved perceived image quality or photorealism, but such relationships must be demonstrated rather than assumed. In particular, reference similarity is neither a necessary nor a sufficient condition for perceived photorealism when physically plausible variations in lighting, viewpoint-dependent appearance, texture realization, or material microstructure are involved.

The high proportion of DISTS and HDR-VDP-2 applications associated with perceptual validation is noteworthy but should be interpreted cautiously because each metric appeared in only four corpus studies. Their evidence base is too small to support a universal ranking against SSIM or LPIPS.

### 4.3. What the Four Metrics Contribute to Rendering Evaluation

#### 4.3.1. SSIM

SSIM was the most prevalent general-purpose target metric in the reviewed corpus and performed strongly for spatially coherent graphics artifacts [7,8]. Its principal limitation is interpretive: high structural similarity can coexist with visible material differences [10] and weak alignment with realism or NVS preference [17,18,19,22]. SSIM is therefore best treated as structural agreement rather than a generic photorealism score. Its high prevalence within the reviewed corpus also makes it useful as a secondary comparison metric because readers can interpret new results against a substantial body of prior work. The present evidence nevertheless argues against using SSIM alone when the experimental manipulation primarily changes texture statistics, subtle visibility, or higher-level plausibility rather than local structure.

#### 4.3.2. LPIPS

LPIPS is conceptually attractive for learned rendering because deep-feature distance can capture perceptually meaningful differences beyond pixels [3]. Yet its human alignment was also content- and task-dependent [17,19,20,22]. It is most defensible when the target construct is feature-level perceptual similarity, not when realism or credibility is assumed without independent validation. In particular, lower LPIPS should be described as greater similarity in the learned feature representation, not automatically as greater realism. This wording becomes important when a plausible rendering departs from the exact reference in viewpoint-dependent highlights, texture realization, or other appearance details.

#### 4.3.3. DISTS

DISTS explicitly combines structure and texture [4], a useful property for materials and neural-rendering artifacts. It performed among the stronger full-reference measures in some human-rated NeRF studies [18,19], but only four applications were identified. DISTS should therefore be viewed as promising complementary evidence rather than a broadly validated replacement for established metrics.

#### 4.3.4. HDR-VDP-2

HDR-VDP-2 uniquely models luminance-dependent visibility and masking [1]. Graphics-artifact studies showed that it can complement SSIM, particularly for some small or scattered visible differences [7,8]. Its HVS basis also makes it relevant to HDR and lighting problems, but visibility prediction is not the same as realism: a visible difference may be acceptable, while a small numerical difference may still violate higher-level scene plausibility [5,23].

### 4.4. Human–Metric Agreement Is Conditional on the Perceptual Task

Across RQ3, context dependence was the most consistent finding. Metric performance varied with artifact morphology [7,8], synthetic versus realistic content [19], image alignment [35], temporal presentation [17], and the perceptual construct assessed in observers. Similarity, fidelity, artifact visibility, realism, immersion, and preference are related but distinct outcomes.

Future validation studies should therefore define the observer task explicitly and test objective scores against that specific construct. A correlation with one perceptual outcome should not be generalized automatically to another. Where several perceptual mechanisms may contribute, a multi-metric approach can be more defensible than reliance on a single score, provided each metric has a clear role [20].

### 4.5. Implications for Controlled Evaluation of Rendering Attributes

The cue analysis reinforces this context-specific interpretation. The six attributes used for RQ4 were derived from a previously published cue-based taxonomy of photorealism in 3D-rendered architectural scenes [51] and were applied here as a predefined framework rather than as categories generated from the review corpus. Lighting had 19 direct applications but limited human grounding, indicating that the literature provides stronger evidence for metric use under illumination changes than for the direct prediction of perceived lighting credibility. Contact shadows showed the opposite pattern: only two direct studies were identified, but both included observer-based validation of shadow acne and peter-panning [7,8]. Reflection evidence increased after renewed full-text retrieval, but direct metric–human validation remained largely absent. This contrast between application frequency and human grounding is informative: a cue may be well represented in technical evaluations yet remain perceptually under-validated, or it may be sparsely represented but supported by unusually direct observer evidence.

Texture had moderate Direct/Supportive coverage and some of the strongest material-related human evidence [9,10,37]. Because perceived texture depends on resolution, mapping, gloss, bump structure, illumination, and geometry, a single global structural score may not capture all perceptually relevant differences. Reflection was also moderately represented [16,26,27,38,50], but direct metric–human validation was largely absent, and viewpoint-dependent appearance can complicate pixel-aligned comparisons.

Micro-imperfections and material translucency remain the clearest validation gaps. The available literature does not provide controlled psychophysical evidence showing whether changes in SSIM, LPIPS, DISTS, or HDR-VDP-2 reliably track human judgments when these cues are manipulated independently. Accordingly, the present review does not conclude that SSIM, LPIPS, or the other target metrics fail to capture these attributes; rather, their cue-specific perceptual validity remains unestablished. Reference-based evaluation may also be sensitive to the choice of reference image: a physically plausible change in scratches, roughness irregularity, or subsurface appearance can reduce numerical similarity when that change is absent from the selected reference. Whether such a numerical difference corresponds to reduced perceived realism requires direct observer validation.

### 4.6. Implications for Future Rendering and Photorealism Research

Future rendering studies should use objective metrics as complements to, rather than substitutes for, human evaluation whenever the claim is explicitly perceptual. Small numerical differences should not be overinterpreted without evidence that observers can detect or value them, and metric roles should be reported clearly so that training losses are not presented as independent output validation.

A central research gap identified by this review is the lack of controlled experiments that test whether changes in objective metric scores track changes in human judgments when individual photorealism-related attributes are manipulated independently. Existing studies rarely isolate lighting credibility, contact shadows, texture credibility, reflection credibility, surface micro-imperfections, or material translucency while holding other scene factors constant. The clearest validation gaps concern reflection, surface micro-imperfections, and material translucency.

To make cue-isolated validation reproducible, future studies can use the following step-by-step protocol:

1. **Predefine the target cue and manipulation.** Select one rendering attribute—for example, lighting credibility, contact shadows, texture credibility, reflection credibility, surface micro-imperfections, or material translucency—and specify in advance how it will be manipulated. Establish a controlled reference rendering and at least two graded variants that alter the target cue while remaining visually plausible.
2. **Construct controlled stimuli.** Use multiple scenes, objects, or materials for each cue to reduce dependence on a single stimulus. Camera position, geometry, composition, image resolution, post-processing, and all non-target rendering parameters should remain fixed so that the intended cue is the principal systematic difference between variants.
3. **Standardize presentation conditions.** Present stimuli under documented and, where feasible, calibrated display conditions. Display resolution and scaling, viewing distance, ambient illumination, image size, dynamic-range settings, and presentation duration should be held constant or explicitly modeled as experimental factors.
4. **Define the observer protocol before data collection.** Specify the target observer population, expertise criteria, sample-size rationale, exclusion criteria, and perceptual task. Stimulus order should be randomized, left–right placement should be counterbalanced for pairwise comparisons, and practice trials should precede the experimental trials. Repeated trials can be included to quantify within-observer consistency.
5. **Collect cue-specific and global perceptual judgments.** Observers should evaluate the manipulated attribute directly, for example through randomized pairwise comparisons or bounded rating scales, and a separate overall quality or realism judgment can be collected when relevant. Cue-specific and global judgments should remain analytically distinct because they represent different perceptual constructs.
6. **Compute objective metrics using a fixed pipeline.** HDR-VDP-2, LPIPS, DISTS, and SSIM should be computed against the same predefined reference images using fixed image preprocessing, alignment, resolution, dynamic-range handling, metric implementation, and parameter settings. Metric versions and processing choices should be reported sufficiently for replication.
7. **Test metric–human agreement separately for each cue.** Analysis should examine whether metric changes reproduce the direction and ordering of observer judgments and should quantify metric–human association with statistical methods appropriate to the experimental design. Results should be reported by cue and, where possible, by scene or stimulus, with uncertainty estimates and disagreement cases retained rather than pooling fundamentally different visual attributes into a single association.

This design would distinguish sensitivity to reference-image differences from sensitivity to perceptually meaningful changes in a specific rendering attribute and would allow the strengths and failure modes of individual metrics to be compared under controlled conditions.

Spatially resolved outputs and complementary multi-metric reporting may also preserve information that is lost in one global score, especially for localized, material-dependent, or heterogeneous rendering artifacts.

### 4.7. Strengths and Limitations

This review has several strengths. Metric-specific searches were conducted across Scopus, Web of Science Core Collection, and IEEE Xplore from database inception to 17 August 2026, followed by renewed full-text retrieval through 19 August 2026. The final strict corpus comprised 283 application studies, with explicit verification of independent output-evaluation use and a clear distinction between direct metric–human validation and parallel subjective evidence. Additional strengths include domain-based methodological appraisal of the 13 direct-validation studies, graded RQ4 evidence that avoided false equivalences such as ambient occlusion versus contact shadows and participating-media scattering versus material translucency, and an availability sensitivity analysis for apparently eligible reports that remained unavailable. Throughout the extraction, full report-level evidence was kept separate from indexed or abstract-level evidence rather than treating apparent eligibility as equivalent to inclusion.

Several limitations remain. Forty-one of the 351 reports sought for retrieval (11.7%) could not be fully assessed: 40 appeared potentially eligible based on indexed evidence, while the target-metric role of one report could not be verified. These reports remained unavailable despite renewed institutional-authentication attempts and publisher/DOI, repository, and exact-title searches. The extreme-case sensitivity analysis was intended only to test the stability of broad corpus-level prevalence findings under an intentionally conservative inclusion scenario. It indicated that assuming all 40 apparently eligible reports were included would not reverse the principal SSIM/LPIPS prevalence ranking or the dominance of neural rendering/NVS. Because only indexed metadata and abstracts were available for these reports, however, this analysis cannot determine their full eligibility, metric role, cue-specific classification, or human-validation status and therefore cannot exclude availability or selection bias for finer application categories or perceptual-validation outcomes. Primary title/abstract screening and initial extraction were conducted by one reviewer; however, a second assessor independently verified all 27 report-level exclusions, all 23 human-evidence studies, the final RQ4 coding, and the methodological appraisal of the 13 direct-validation studies. Any differences identified during verification were resolved by consensus before the final dataset was locked. Because the verification procedure was not designed as a formal inter-rater reliability study, disagreement frequencies were not quantified and no inter-rater statistic was calculated. The absence of fully duplicated title/abstract screening of all 680 records remains a source of possible selection bias and screening error. The evidence was also highly heterogeneous across tasks, datasets, metrics, human-study designs, and perceptual outcomes, precluding meta-analysis.

The search strategy was limited to Scopus, Web of Science Core Collection, and IEEE Xplore, and ACM Digital Library was not queried as a separate identification source. Although ACM publications indexed in the selected multidisciplinary databases could be retrieved, ACM-only records could not enter the search directly. This limitation is most consequential for older conference-based computer-graphics and graphics-artifact literature and may have reduced the historical coverage of such studies. The corpus was also strongly skewed toward recent neural rendering: 239 of 283 studies were published in 2024–2026, and 175 belonged to neural rendering/NVS/3D reconstruction. Corpus-level prevalence should therefore be interpreted as reflecting contemporary indexed learned-rendering research more strongly than the complete historical literature on computer-generated imagery. In addition, the review was intentionally limited to four target metrics; other full-reference, no-reference, or task-specific measures may perform better in particular contexts [17,23,36].

Finally, RQ4 mapped existing studies onto six visual attributes that were defined before the final synthesis and were grounded in the previously published cue framework [51], but most included studies were not originally designed to test that framework directly. Direct, Supportive, and Adjacent categories reduced overinterpretation, yet applicability judgments remain partly interpretive. Sparse evidence for micro-imperfections and translucency should therefore be read primarily as a research gap rather than as evidence that the reviewed metrics are ineffective.

## 5. Conclusions

This systematic review identified a substantial imbalance between metric adoption and perceptual validation in rendering research. Across 283 application studies, SSIM and LPIPS were widely used, particularly in recent neural rendering, whereas only 23 studies contained relevant human evidence and 13 directly tested metric–human agreement. Routine benchmark use should therefore not be interpreted as evidence that a metric is a validated predictor of perceived quality, realism, or credibility.

The direct-validation evidence further showed that metric–human agreement depends on content, artifact type, alignment, viewing conditions, and the perceptual construct being assessed. Cue-specific evidence was similarly uneven: lighting was widely represented but had limited human grounding; contact-shadow evidence was sparse but supported by direct observer validation; texture and reflection had moderate application evidence but limited direct validation; and surface micro-imperfections and material translucency remained the clearest validation gaps. The available evidence therefore supports the context-specific, complementary use of objective metrics, with human validation required whenever conclusions extend from reference similarity to claims about perception.

## Figures and Tables

**Table 1:** Overall characteristics of the strict application corpus (n=283).

Characteristic	*n*	%
**Publication period**		
2010–2020	14	4.9
2021–2023	30	10.6
2024–2026	239	84.5
Target metric *		
SSIM	228	80.6
LPIPS	147	51.9
HDR-VDP-2	4	1.4
DISTS	4	1.4
**Metric configuration**		
SSIM only	130	45.9
LPIPS only	54	19.1
SSIM + LPIPS	91	32.2
SSIM + HDR-VDP-2	4	1.4
SSIM + LPIPS + DISTS	2	0.7
DISTS only	1	0.4
SSIM + DISTS	1	0.4

* Metric frequencies are non-exclusive.

**Table 2:** Conceptual comparison of the four target metrics.

Metric	Principal Representation	Primary Perceptual Construct	Main Output Interpretation	Main Advantage	Principal Limitation for Rendering Realism
SSIM	Local luminance, contrast, and structure	Structural similarity	Higher similarity indicates greater structural agreement	Simple, interpretable, widely implemented	Does not explicitly model HVS visibility or higher-level realism
LPIPS	Deep neural-network feature distance	Learned perceptual similarity	Lower distance indicates greater learned-feature similarity	Often sensitive to perceptually meaningful feature differences	Alignment with human judgment depends on content and perceptual task
DISTS	Deep structure + texture statistics	Structural and texture similarity	Lower distance indicates greater perceptual similarity	Explicitly distinguishes structure from texture-related variation	Very limited rendering-specific application base
HDR-VDP-2	Calibrated HVS model	Difference visibility and perceptual quality	Visibility map and quality prediction	Models luminance adaptation, masking, and threshold visibility	Requires meaningful display/luminance assumptions and does not encode semantic plausibility

**Table 3:** Target-metric use by primary application family.

Primary Application Family	*n*	SSIM	LPIPS	HDR-VDP-2	DISTS
Neural rendering/NVS/3D reconstruction	175	139	107	0	0
Material/appearance/lighting	26	20	12	0	0
IQA/artifact & perceptual assessment	21	18	6	3	4
Avatar/digital-human rendering	19	13	11	0	0
Traditional rendering/enhancement/optimization	20	19	5	1	0
VR/display/immersive rendering	17	14	5	0	0
Synthetic data/simulation/application-specific rendering	5	5	1	0	0
Total	283	228	147	4	4

RQ2 application families are mutually exclusive. Metric counts are non-exclusive because individual studies could report more than one target metric.

**Table 4:** Representative direct human-validation evidence.

Study	Context	Metric(s)	Human Evidence	Main Finding
Čadík et al. [5]	Graphics artifacts	SSIM, HDR-VDP-2	35 observers, spatial artifact maps	Important failures for luminance/contrast and higher-level plausibility
Piórkowski and Mantiuk [7]	Game-engine artifacts	SSIM, HDR-VDP-2	47 observers, ROC/AUC	SSIM strongest overall; HDR-VDP-2 particularly effective for scattered aliasing
Piórkowski et al. [8]	Game-engine artifacts	SSIM, HDR-VDP-2	55 observers, ROC/AUC	Artifact-dependent metric performance replicated
Azari et al. [10]	BTF/material texture	SSIM	20 observers	Very high SSIM did not guarantee perceptual equivalence
Liang et al. [17]	NeRF/NVS	SSIM, LPIPS, DISTS	39 observers	PSNR outperformed SSIM and LPIPS for perceptual ranking
Onuoha et al. [18]	NeRF	SSIM, DISTS	Subjective quality scores	DISTS among stronger correlations; SSIM weaker
Luo et al. [19]	NeRF	SSIM, LPIPS, DISTS	MOS, N = 86	DISTS among stronger FR metrics; content dependence
Cutler et al. [22]	Photorealistic avatars	SSIM, LPIPS	Multidimensional QoE	Weak realism correlation for SSIM/LPIPS
Cao et al. [23]	HDR rendering	SSIM/comparators	Four human-rated HDR datasets	HDR-specific perceptual models outperformed generic alternatives
Ghildyal et al. [35]	Non-aligned NVS	SSIM/comparators	Five expert annotators	Misalignment motivates NVS-specific perceptual comparison
Trioux et al. [36]	Avatar animation	SSIM/comparators	Subjective MOS	Objective–subjective agreement moderate at best

**Table 5:** Evidence relevant to the six rendering attributes.

Rendering Attribute	Direct	Supportive	Adjacent	Human-Linked *
Lighting credibility	19	0	0	2
Contact shadows	2	0	1	2
Texture credibility	7	4	0	3
Reflection credibility	8	1	0	1
Surface micro-imperfections	1	2	0	0
Material translucency	2	0	0	0

* Human-linked counts are subsets of the Direct/Supportive evidence counts and are not additive.

## Data Availability

All data generated during this systematic review are provided within the article and its Supplementary Materials. The supplementary workbook contains the complete search strategies, included-study characteristics with manuscript reference numbers, human-validation coding and methodological appraisal, cue-specific evidence, reports not retrieved with availability sensitivity accounting, and report-level exclusions.

## Supplementary Materials

The following supporting information can be downloaded at https://www.mdpi.com/article/10.3390/jimaging12100500/s1: Table S1: Complete database search strategies and search yields; Table S2: Characteristics of the 283 studies included in the strict application corpus; Table S3: Human-validation evidence and methodological appraisal (n = 23); Table S4: Cue-specific evidence relevant to RQ4; Table S5: Final reports not retrieved (n = 41); Table S6: Reports excluded after report-level assessment (n = 27). The completed PRISMA 2020 checklist is provided as a separate reporting file.
