# Supplementary material for: HDR-VDP-2, LPIPS, DISTS, and SSIM for Evaluating Computer-Generated Renderings: A Systematic Review of Use and Perceptual Validation
Source: J Imaging. 2026 Oct 10;12(10):500. doi: 10.3390/jimaging12100500 (PMC13706470; doi:10.3390/jimaging12100500)
Supplement: Supplementary file 1 [file jimaging-12-00500-s001.zip › PRISMA_2020_Checklist_new.pdf]

PRISMA 2020 Checklist

Manuscript: Objective Image-Quality Metrics for Evaluating Computer-Generated Renderings: A Systematic Review of HDR-VDP-2, LPIPS, DISTS, and SSIM

Manuscript ID: jimaging-4569862 | OSF retrospective registration: https://doi.org/10.17605/OSF.IO/GWMNP

Section and Topic	Item #	Checklist item	Location where item is reported
**TITLE**			
Title	1	Identify the report as a systematic review.	Title page, p. 1 (identified as a Systematic Review).
**ABSTRACT**			
Abstract	2	See the PRISMA 2020 for Abstracts checklist.	Abstract, p. 1.
**INTRODUCTION**			
Rationale	3	Describe the rationale for the review in the context of existing knowledge.	Section 1, Introduction, pp. 1-3.
Objectives	4	Provide an explicit statement of the objective(s) or question(s) the review addresses.	Section 1, Introduction (review objective and RQ1-RQ4), pp. 3-4.
**METHODS**			
Eligibility criteria	5	Specify the inclusion and exclusion criteria for the review and how studies were grouped for the syntheses.	Sections 2.2 and 2.7, pp. 4 and 6-7.
Information sources	6	Specify all databases, registers, websites, organisations, reference lists and other sources searched or consulted to identify studies. Specify the date when each source was last searched or consulted.	Section 2.3, p. 5 (Scopus, Web of Science Core Collection, IEEE Xplore; search and retrieval dates also reported).
Search strategy	7	Present the full search strategies for all databases, registers and websites, including any filters and limits used.	Section 2.4, p. 5; complete database-specific strategies and yields in Supplementary Table S1.
Selection process	8	Specify the methods used to decide whether a study met the inclusion criteria of the review, including how many reviewers screened each record and each report retrieved, whether they worked independently, and if applicable, details of automation tools used in the process.	Section 2.5, pp. 5-6 (deduplication, screening, retrieval, report-level assessment, reviewer roles, and verification).
Data collection process	9	Specify the methods used to collect data from reports, including how many reviewers collected data from each report, whether they worked independently, any processes for obtaining or confirming data from study investigators, and if applicable, details of automation tools used in the process.	Sections 2.5-2.6, pp. 5-6 (reviewer roles, verification, and standardized data extraction).
Data items	10a	List and define all outcomes for which data were sought. Specify whether all results that were compatible with each outcome domain in each study were sought (e.g. for all measures, time points, analyses), and if not, the methods used to decide which results to collect.	Section 2.6, p. 6 (target-metric outputs and human perceptual outcomes/constructs sought).
	10b	List and define all other variables for which data were sought (e.g. participant and intervention characteristics, funding sources). Describe any assumptions made about any missing or unclear information.	Sections 2.6-2.7, pp. 6-7 (bibliographic, domain/task, metric-role, human-evidence, and six cue-specific variables).
Study risk of bias assessment	11	Specify the methods used to assess risk of bias in the included studies, including details of the tool(s) used, how many reviewers assessed each study and whether they worked independently, and if applicable, details of automation tools used in the process.	Section 2.8, p. 7. No single clinical-style risk-of-bias tool was applied across all 283 heterogeneous studies; the 13 direct metric-human validation studies received a six-domain methodological appraisal. Details in Supplementary Table S3.
Effect measures	12	Specify for each outcome the effect measure(s) (e.g. risk ratio, mean difference) used in the synthesis or presentation of results.	Not applicable as a common effect-measure synthesis. Counts, percentages, and structured narrative evidence were used; Section 2.9, pp. 7-8.
Synthesis methods	13a	Describe the processes used to decide which studies were eligible for each synthesis (e.g. tabulating the study intervention characteristics and comparing against the planned groups for each synthesis (item #5)).	Sections 2.7 and 2.9, pp. 6-8 (RQ-specific grouping/coding and synthesis eligibility rules).

Section and Topic	Item #	Checklist item	Location where item is reported
	13b	Describe any methods required to prepare the data for presentation or synthesis, such as handling of missing summary statistics, or data conversions.	Sections 2.6-2.9, pp. 6-8 (standardized extraction and coding for descriptive synthesis; no meta-analytic data conversion was performed).
	13c	Describe any methods used to tabulate or visually display results of individual studies and syntheses.	Section 2.9, pp. 7-8; Tables 1-5; Figures 1-3; Supplementary Tables S1-S6.
	13d	Describe any methods used to synthesize results and provide a rationale for the choice(s). If meta-analysis was performed, describe the model(s), method(s) to identify the presence and extent of statistical heterogeneity, and software package(s) used.	Section 2.9, pp. 7-8 (descriptive statistics and structured narrative synthesis; rationale for no meta-analysis).
	13e	Describe any methods used to explore possible causes of heterogeneity among study results (e.g. subgroup analysis, meta-regression).	No formal subgroup analysis or meta-regression was performed. Context-dependent variation was handled through predefined application families, evidence grades, and narrative synthesis; Sections 2.7 and 2.9, pp. 6-8.
	13f	Describe any sensitivity analyses conducted to assess robustness of the synthesized results.	Section 2.9, p. 8 (extreme-case availability sensitivity analysis for 40 apparently eligible but unretrieved reports).
Reporting bias assessment	14	Describe any methods used to assess risk of bias due to missing results in a synthesis (arising from reporting biases).	No formal reporting-bias assessment was performed because no common effect estimate or homogeneous outcome was synthesized; Section 2.9, p. 8. Availability bias was examined separately by sensitivity analysis.
Certainty assessment	15	Describe any methods used to assess certainty (or confidence) in the body of evidence for an outcome.	No single overall certainty-of-evidence grade was used. Confidence in RQ3 was characterized by study-level methodological appraisal and evidence directness; RQ4 used Direct/Supportive/Adjacent grades; Sections 2.8-2.9, pp. 7-8.
**RESULTS**			
Study selection	16a	Describe the results of the search and selection process, from the number of records identified in the search to the number of studies included in the review, ideally using a flow diagram.	Section 3.1, pp. 8-9; PRISMA flow diagram, Figure 1, p. 9.
	16b	Cite studies that might appear to meet the inclusion criteria, but which were excluded, and explain why they were excluded.	Section 3.1, pp. 8-9; all 27 report-level exclusions with reasons in Supplementary Table S6.
Study characteristics	17	Cite each included study and present its characteristics.	Section 3.2, pp. 9-10; Table 1, p. 10; all 283 included studies and characteristics in Supplementary Table S2; references [5-290].
Risk of bias in studies	18	Present assessments of risk of bias for each included study.	Section 3.5.6, p. 14; study-level domain judgments for the 13 direct-validation studies in Supplementary Table S3. No universal risk-of-bias assessment was applied to all 283 studies (see Section 2.8).

PRISMA 2020 Checklist

Section and Topic	Item #	Checklist item	Location where item is reported
Results of individual studies	19	For all outcomes, present, for each study: (a) summary statistics for each group (where appropriate) and (b) an effect estimate and its precision (e.g. confidence/credible interval), ideally using structured tables or plots.	Sections 3.5.1-3.5.5, pp. 12-14; representative direct human-validation results in Table 4, p. 14; complete study-level evidence in Supplementary Tables S2-S4. No common effect estimates/precision were synthesized.
Results of syntheses	20a	For each synthesis, briefly summarise the characteristics and risk of bias among contributing studies.	Sections 3.2-3.6, pp. 9-17; methodological-appraisal results in Section 3.5.6, p. 14; Tables 1-5 and Supplementary Tables S2-S4.
	20b	Present results of all statistical syntheses conducted. If meta-analysis was done, present for each the summary estimate and its precision (e.g. confidence/credible interval) and measures of statistical heterogeneity. If comparing groups, describe the direction of the effect.	No meta-analysis was performed. Descriptive synthesis results are reported in Sections 3.2-3.6, pp. 9-17, and Tables 1, 3, and 5.
	20c	Present results of all investigations of possible causes of heterogeneity among study results.	No formal heterogeneity analysis was performed. Context-dependent differences are reported in Sections 3.5.1-3.5.5, pp. 12-14, and interpreted in Sections 4.3-4.5, pp. 18-19.
	20d	Present results of all sensitivity analyses conducted to assess the robustness of the synthesized results.	Section 3.1, pp. 8-9 (availability sensitivity analysis and extreme-case augmented-corpus results).
Reporting biases	21	Present assessments of risk of bias due to missing results (arising from reporting biases) for each synthesis assessed.	No formal reporting-bias assessment was conducted. Availability-related bias is addressed by the sensitivity analysis in Section 3.1, pp. 8-9, and limitations in Section 4.7, pp. 20-21.
Certainty of evidence	22	Present assessments of certainty (or confidence) in the body of evidence for each outcome assessed.	No formal overall certainty grade was assigned. Confidence is reported through the RQ3 methodological appraisal (Section 3.5.6, p. 14; Supplementary Table S3) and RQ4 evidence grades (Section 3.6, pp. 14-17; Supplementary Table S4).
**DISCUSSION**			
Discussion	23a	Provide a general interpretation of the results in the context of other evidence.	Sections 4.1-4.5, pp. 17-19; Section 5, Conclusions, p. 21.
	23b	Discuss any limitations of the evidence included in the review.	Section 4.7, pp. 20-21 (limitations of the included evidence, including heterogeneity, unavailable reports, and sparse cue-specific validation).
	23c	Discuss any limitations of the review processes used.	Section 4.7, pp. 20-21 (review-process limitations, including single-reviewer primary screening/extraction and database coverage).
	23d	Discuss implications of the results for practice, policy, and future research.	Sections 4.5-4.6, pp. 19-20; Section 5, Conclusions, p. 21.
**OTHER INFORMATION**			

PRISMA 2020 Checklist

Section and Topic	Item #	Checklist item	Location where item is reported
Registration and protocol	24a	Provide registration information for the review, including register name and registration number, or state that the review was not registered.	Section 2.1, p. 4. Retrospectively registered on the Open Science Framework (OSF) on 2 September 2026: https://doi.org/10.17605/OSF.IO/GWMNP .
	24b	Indicate where the review protocol can be accessed, or state that a protocol was not prepared.	Section 2.1, p. 4. The review was not prospectively registered; the retrospective OSF registration documents the finalized review methods: https://doi.org/10.17605/OSF.IO/GWMNP .
	24c	Describe and explain any amendments to information provided at registration or in the protocol.	Not applicable. There was no prospective registration/protocol to amend; the OSF registration was created retrospectively after completion following editorial request (Section 2.1, p. 4).
Support	25	Describe sources of financial or non-financial support for the review, and the role of the funders or sponsors in the review.	Funding statement, p. 21: no external funding.
Competing interests	26	Declare any competing interests of review authors.	Conflicts of Interest statement, p. 22.
Availability of data, code and other materials	27	Report which of the following are publicly available and where they can be found: template data collection forms; data extracted from included studies; data used for all analyses; analytic code; any other materials used in the review.	Supplementary Materials and Data Availability Statement, pp. 21-22; Supplementary Tables S1-S6 contain search strategies, study-level extraction/coding, appraisal, cue evidence, unretrieved reports, and exclusions.

From: Page MJ, McKenzie JE, Bossuyt PM, Boutron I, Hoffmann TC, Mulrow CD, et al. The PRISMA 2020 statement: an updated guideline for reporting systematic reviews. BMJ 2021;372:n71. doi: 10.1136/bmj.n71. This work is licensed under CC BY 4.0. To view a copy of this license, visit https://creativecommons.org/licenses/by/4.0/
